# Chicago Medical Society

**Published:** 1874-11

**Authors:** 

**Affiliations:** Reporter pro tem.


**Chicago Medical Society.** *Transactions at the Meeting of Aug.* 17, 1874. Dr. GRAHAM, Reporter *pro tem.*

The President, Dr. Quine, in the Chair.

Dr. Stillians presented a specimen of intestine, showing intussusception of the colon at five different points, three of which were well marked. The patient was a boy five years old. The only symptoms at first were constipation and colicky pains, for which anodynes and cathartic enemata were given. There was afterwards some flatulence, and a tender point was discovered below and to the right of the umbilicus. A very small quantity of feces was passed at two or three different times after giving the injections. During the second day the skin became hot and the pulse rapid and feeble, which symptoms continued and became more prominent, death ensuing that night.

In answer to a question, the Dr. stated that there had not been any vomiting or discharge of blood from the bowels. Dr. Dyas said it was very unusual not to have these symptoms, and that there was generally a tumor on the right side. Death was often caused by the shock to the system, without any symptoms of inflammation being present. Purgatives should not be given in such cases, but enemata of simple water and opiates should be chiefly relied on.

Dr. A. H. Foster read a report of a case of hydrophobia.

On motion, the discussion of this paper was deferred till the next meeting.

Dr. H. M. Lyman read the following report of his observations on three cases of diabetes mellitus, occurring in the County Hospital during his term of service :

*Diabetes Mellitus.*—Three cases of this disease were under treatment. One died ; another left the hospital, after remaining three months under treatment ; the third was still in the house at the close of my term of service. He was a man in middle life, whose case presented no unusual symptoms. The boy who left us was of Swedish parentage, æt. 17 years; admitted to the hospital April 18, 1874. Had never noticed any failure of health till about March 1, when he began to experience great thirst, dryness of the skin, frequent and copious urination, dull aching in the legs, progressive weakness, a ravenous appetite, and some indistinctness of vision. On admission, his pulse, temperature and respiration were normal, but the skin was dry and desquamating, and his tongue was red and dry in the centre. During the first twenty-four hours after admission, the patient voided twelve pints of urine, (sp. gr. 1032), which gave a decidedly saccharine reaction with Fehling's test. He was ordered the most nitrogenous diet which our somewhat limited resources could supply; was douched with cold water every morning, and took once every four hours a scruple of bromide of potassium, with an equal quantity of chlorate of potassium. He had ten grains of Dover's powder every night. April 24, voided seventeen and a half pints of urine.

The quantity gradually diminished till May 3, when it was only nine pints. At this point it remained stationary until May 12, when the treatment was changed, in order to make trial of a recent German theory of the disease, and the patient took nothing but five grains of carbolic acid three times daily. This treatment proved unsuccessful, for the quantity of urine rapidly increased. May 22 it was fourteen pints; June 1, sixteen pints; June 16, eighteen pints. Medicine now seemed to have very little control over the disease; but when the uncommonly hot weather in July began to stimulate the functions, the urinary discharge was rapidly diminished, and when the patient left the hospital, July 18, he voided six pints *per diem*. In every other particular, however, his condition was unchanged.

The diabetic patient who died under treatment was an Irish laborer, æt. 22 years, admitted Feb. 11, 1874. For about six months he had been losing flesh and strength. Had experienced a slight tracheal irritation, but did not actually begin to cough till three weeks before entering the hospital. On admission, he was very much emaciated; his face was flushed; skin harsh and dry; tongue clean; appetite good; bowels costive; urine copious (sp. gr. 1020) and saccharine. A slight cough attracted attention to his lungs: respiration was largely abdominal, and the respiratory sounds were exaggerated.

He was ordered to take cod liver oil, with citrate of iron and quinine. The quantity of urine gradually diminished from fifteen pints *per diem*, till May 5, when it was only eight pints.

He was now ordered to take carbolic acid instead of iron and quinine, but no benefit was derived from the change. As the weather grew warm he voided less water, but the disease progressed, and he died July 14

Autopsy, thirty hours after death. Extreme dryness of all the tissues was remarked. The brain was very anæmic, but otherwise healthy. The condition of the left lung was normal, but the lower lobe of the right lung was tubercular, and contained several suppurating cavities. The liver and spleen were of the usual weight, but were filled with masses of tubercle, which had in several places degenerated into tubercular abscesses. A similar abscess, as large as a pea, was found upon the greater convexity of the left kidney. The other viscera presented no unusual appearance.

Dr. J. S. Knox gave the details of a case of uterine disease, in which there was sloughing of the entire cervix uteri. The patient had been under treatment for twenty-seven years by many different physicians. Had submitted to leeching, scarification, cautery, fomentations, douches, paintings with iodine and chromic acid, and had tried "pessaries enough to support the womb of time through all eternity." She came under the Dr.'s care by applying for readjustment of a Hodge pessary, which she had worn for a long time.

It was removed on account of the inflammation, which it seemed to have produced, and emollient treatment was pursued several weeks, when the sloughing took place. The rapid healing of the stump, and the absence of every sign or symptom which would cause a suspicion of malignant disease, led the Dr. to conclude that the sloughing was the result of diminished vascularity and vitality, due to the many recurring inflammations, and the abundant treatment of previous years.

Dr. W. E. Clark exhibited the sac of an ovarian tumor which weighed fifty pounds. The Dr. had removed the tumor this afternoon. Both ovaries were diseased, and there was also a fibroid tumor of the uterus.

A motion was made and passed, after considerable discussion, to appoint a committee of three to co-operate with similar committees from the Society of Physicians and Surgeons, and the College of Pharmacy, in considering the relations of physicians and pharmacists, and to consider and report any measures thought necessary to check the prescribing of proprietary medicines.

Drs. A. H. Foster, Stillians and C. W. Earle were appointed such committee.

On motion, Society adjourned.

*Transactions at Meeting of Sept.* 7, 1874. Reported by WILL T. MONTGOMERY, M.D.

Chicago Medical Society met as usual in the parlor of the Gault house, the President, Dr. Quine, in the Chair.

The discussion of Dr. Foster's paper on hydrophobia, read at the last meeting, having been postponed, first occupied the attention of the Society. Dr. Foster, upon further inquiry, had learned that the dog had been acting queerly for several days previous to the time of biting the child. There was nothing peculiar in the appearance of the body after death.

Dr. Earle hoped that the question of virus or non-virus, upon which the profession are divided in opinion, would be considered.

Dr. N. S. Davis had not heard the paper read, and was not prepared to discuss it. He had never seen but one case of hydrophobia, in a practice of nearly forty years, and in that case it was not possible to trace the origin of the disease to any bite. The patient had all of the symptoms and post-mortem appearances of the disease. His impression had been that we may get all the phenomena of the disease without infection from bites.

Dr. Holmes had seen a case about twenty years ago, in the Massachusetts General Hospital. He first saw the patient, a little girl, about twelve hours after she had been bitten by a rabid dog. The wound was thoroughly cauterized at this time, and the patient was taken to her home. In about a week she was brought back to the hospital, with well marked hydrophobia, and soon died. The least draft of air upon the surface, even the breath of the attendant, was sufficient to excite spasm in this case. He believed the poison to be specific, and thought it possible for a patient to be bitten without knowing it.

Dr. T. D. Fitch had never seen a genuine case of hydrophobia, but had recently seen a spurious case in a man who had been bitten by a healthy cat. The patient soon began to improve upon the use of nervous sedatives, but had a relapse from reading the late discussion of the disease in the newspapers.

Dr. E. F. Ingals. It seems from the literature of the subject, that this disease has been known almost since the time of Adam. It seems to have been most frequently met with in temperate climates, and is said never to occur in Egypt, and other portions of Africa; in the hottest portions of South America; in Greenland, Labrador and Iceland. It rarely occurs in the Southern portion of Turkey, and seems less frequent in the Southern than in the Northern States of this country. The people have the impression that it seldom occurs except in summer, but this view is incorrect. Out of 100 cases of hydrophobia, in which the time of receiving the bite is recorded, we find, spring twenty-five; summer thirty-five; autumn thirteen, and winter twenty-seven. The disease is common to the canine and feline races, and has frequently occurred in horses, cattle and swine, as the result of inoculation. One rather doubtful case is reported as having been produced by the bite of a cock. Hydrophobia never occurs spontaneously in man, in whom it is a rare disease. In France, it is said, one case occurs yearly to every 1,500,000 inhabitants. In dogs, the disease is doubtless spontaneous in some instances, but is generally the result of inoculation. For instance, a ship arrived in the port of Havana, after a long voyage of several months, at the time that hydrophobia was rife in the city. The dogs on board soon took the disease, though they had not been on shore. Prediposing causes to this affection in dogs are said to be putrid aliment, bad and deficient supply of water, and extreme heat and cold. Thousands of dogs have been exposed to the two former causes without taking it, and rarely or never can a case of this disease be traced to such causes, so that it is doubtful whether there is ground for the belief. Extreme heat and cold produce debility, which render the dog more liable to the disease. Out of 133 cases the bite had been given by a dog in one hundred and five; wolf in twenty; cat in five. During the period of incubation, which lasts from ten days to six months, and more, it is stated by good authority, that an eruption appears beneath the tongue, between the third and fifteenth day. In 235 cases observed, with reference to the period of incubation, in forty-eight cases it was from ten to thirty days; in one hundred and fifty-nine cases, thirty to sixty days; in seventy-five cases, sixty to ninety days; in thirty-one cases, three to six months; in thirteen cases, six to nineteen months; in one case, three years and four months; in one case, four years, and in another, five years and six months. Not more than one-third of the persons bitten by rabid animals have hydrophobia. There is not a single authentic case of recovery.

In the treatment of this disease scutellaria lateriflora was much used in the early part of this century. Cauterizing the vesicles found beneath the tongue, alcohol, opium, and introduction of water, have been recommended.

Dr. Powell suggested tracheotomy as a means of prolonging and perhaps saving life, as the direct cause of death is usually spasm of the glottis.

Dr. Ingals had read of cases in which this had been resorted to without success.

Dr. Van Buren had seen a case of snake-bite, in which alcohol seemed to neutralize the poison. The man was drunk when the snake bit him, yet was given large doses of whisky and recovered. He did not know but alcohol in large quantities might neutralize the poison of hydrophobia also.

A paper by Dr. W. T. Montgomery, which had been announced for the evening, was next called for and read. The paper was a collation from a record of 112 cases of typhoid fever. It was moved and carried that the paper be accepted and published with the proceedings of the Society. The discussion of the paper was postponed until the next meeting.

*Transactions at Regular Meeting held September* 21, 1874.

Chicago Medical Society met in regular session in the parlor of the Gault House. In the absence of the President, Vice-President Paoli was called to the Chair.

The discussion of Dr. Montgomery's paper on Typhoid Fever was first in order of business. Dr. Danforth inquired as to the mortality of the cases under consideration. Dr. Montgomery stated that there were 20 of the 112 cases fatal. Dr. Danforth had recently met a practitioner who had not lost a case in a practice of forty years, and thought he ought to be appointed physician to the hospital. Dr. T. D. Fitch had been in the habit of administering veratrum in mucilaginous drinks, in the early stages of this fever, with good effect. It had not been uncommon with him to find constipation in the beginning of the disease. He had imagined that carbonate of ammonia had a beneficial effect as an antiseptic, and in preventing heart-clot in the late stages of the fever. Dr. Hutchinson thought, as the paper dwelt more particularly upon the use of the thermometer as an auxiliary in the diagnosis and prognosis of this disease, the discussion should be limited to this. Dr. T. D. Fitch had not had much experience with the thermometer in typhoid, but so far as he had used it, had found it a valuable aid.

Dr. Paoli thought it was remarkable that a physician should never have a death from this disease, but thought the mortality much greater some years than others. One year, while he was in Stockholm, the mortality there was about forty per cent. He considered the thermometer valuable in diagnosis and prognosis, but its use was not new. It had been used in Vienna in this disease as much as fifty years ago.

Dr. T. D. Fitch thought we should not entirely ignore the statement of some physicians that they never lose a case of typhoid. It may be a coincidence. He did not remember when he had lost a case.

Dr. Danforth presented microscopical specimens of two ovarian cysts. The first was from a tumor of long standing, removed about a month ago by Dr. Wm. E. Clark. It was for a long time doubtful as to what it was. He was requested to examine some of the fluid from it, and found Eichwald's gorged granules and crystals of colesterin. He had never found either of these from any except an ovarian cyst, and he had examined the contents of a number of these cysts and never failed in but one instance to find the granules, and in that he was not sure that the fluid came from a cyst. These granules are believed to be degenerated cells. The granules and crystals of colesterin were both clearly shown in this specimen.

The second specimen was of colloid cells form, removed by Dr. John E. Owens about six months ago. The tumor in this case was not of so long standing. These cells are found in the early development of ovarian cysts, and the granules in the later.

Dr. Bridge inquired as to the difference between colloid and cancer cells, and if the former were malignant.

Dr. Danforth. The difference is, the colloid cells are all after the same type; cancer cells may have any form. If a morbid growth presents cells all of a certain type, it is not malignant.

Dr. Steele reported the following case for Dr. Quine, and presented specimen of cyst:

Regina W., aged 27, unmarried, has menstruated regularly since the sixteenth year of her age. Five years ago her abdomen began to enlarge, and she noticed, also, that pain attended the first two days of the menstrual flow. In August, 1872, after severe muscular exercise, she was seized with pain in the right lumbar region that continued several months, and was accompanied by fever, progressive emaciation, and rapid increase in the size of the abdomen. So burdensome did the enlargement become, that she was incapacitated for work, and was obliged, in May, 1873, to seek relief in the County Hospital. She was extremely emaciated, her skin was sallow, and her features were expressive of anxiety and suffering. From May, 1873, to August of this year, she was tapped six times, the aggregate amount of fluid evacuated being one hundred and sixty pounds. The fluid was of a dark-greenish color at first, very viscid and thick, and contained a copious admixture of pus; but its character improved with each succeeding paracentesis, and the accumulation became more rapid. In two instances the operation was followed by peritonitis. From the first, tonics were employed, and hygienic measures instituted, with a view to improving nutrition; but ovariotomy was then considered impracticable, because of the supposed existence of pulmonary tubercle. The condition of the patient steadily improved, and a pleurisy and pericarditis, under which she had been laboring, got well. A month after the last tapping, the abdomen having again become moderately distended, it was decided, by a consultation between the attending physician and Drs. Byford and Fitch, that an operation was practicable. On the 12th of August, Dr. Quine, assisted by Drs. Fitch and Bogue and the House Staff of the Hospital, performed the operation—making an incision four and a half inches long in the mesian line, and carrying it rapidly through the walls of the abdomen. The trifling hemorrhage from the wound was readily checked. When the peritoneum was cut, the finger of the operator, passed through the wound, failed to discover any adhesions within the circle of its reach. Thirty pounds of fluid were then evacuated by a large trochar, and as the sac was being withdrawn, adhesions to the omentum and right parietes were revealed. Momentary embarrassment was occasioned by a little aggregation of cysts that resembled a kidney very closely in size, shape and color. The omental adhesions, though not very extensive, were exceedingly vascular, and the parietal adhesions were also vascular and very firm. Some parts of the adhering omentum were tied and cut off, and other parts were gently separated from the cyst walls, and left. Some trouble and delay were occasioned by persistent bleeding from the lacerated parietal adhesions, but sufficient time was taken to check all oozing. The pedicle was ligated by transfixion and double ligature, and cut short. The abdominal cavity was carefully sponged out with tepid salt water, and the wound closed with six silver wire sutures. A compress and bandage completed the dressing. After reaction was fairly established, not a single unfavorable symptom appeared, the wound healing perfectly in six days, and the patient being able to sit up in nine days after the operation.

Dr. Gapen reported the following case also for Dr. Quine, and exhibited tumor:

The doctor saw the lady, a woman of 25 years of age, in a consultation, which was necessitated by malposition of the child in parturition. The labor terminated favorably. Subsequently, owing to the removal of the previous attendant from the city, Dr. Quine was recalled. He then learned that the lady had, for seven years, been suffering from bronchitis and gastro-enteritis, and was reduced to a state of extreme emaciation by these affections. At the first visit the irritability of the stomach was so great that the blandest articles of food were retained with difficulty, and only in very small quantities. Vast quantities of gas were being incessantly eructated and passed from the bowels. The patient was also suffering from a harassing cough, with profuse expectoration, and a very distressing tenesmic diarrhœa.

She improved in condition slowly and unsteadily for several months, and finally, at a time when her health had become better than it had been for years, passed temporarily from the Doctor's care. In November, 1873, she returned in consequence of an exceedingly sensitive tumor that occupied the pelvic cavity, and protruded from the vagina when she assumed a standing posture. She was then seven months pregnant, and complained of a return of the old bronchial and gastro-enteric disorders. A cautious digital examination in his office yielding negative results, the Doctor sent the lady home, promising to call the following evening. He did so, finding her well advanced in labor, the shoulder of the child presenting. The presentation was made out with great difficulty, owing to the occupation of the pelvis by a sensitive, fluctuating tumor, and consequent displacement of the uterus,—the os pointing to the umbilicus, and being very high. Chloroform was administered, and the child easily delivered by podalic version.

As the child was being withdrawn, the tumor, which had been no impediment to labor, ascended into the abdominal cavity. The patient made a very tardy recovery, owing to the occurrence of pelvic cellulitis; and before she was able to leave the bed, a diagnosis of ovarian tumor was made.

During the succeeding seven months the lady was tapped three times, the second and third tappings yielding respectively less fluid, and correspondingly less relief, than the preceding. At the third tapping, performed in consultation with Dr. T. D Fitch, only about a quart of fluid was withdrawn. The abdomen of the patient had reached an enormous size, and the gastro-enteric disorder, coupled with constant pelvic distress, contributed to render life miserable and threateningly short. Ovariotomy was decided upon by Drs. Bartlett, Bogue, Fitch, and Quine, as the only means that offered the slightest prospect of relief or long continuance of life. Accordingly, and notwithstanding the extreme feebleness and emaciation of the patient, and the existence of grave gastro-enteric disease, the operation was performed by Dr. Quine, assisted by Drs. Bogue, T. D. Fitch, Stillians, and myself. The operation was tedious and very difficult. The abdominal incision extended from an inch and a half above the umbilicus to within an inch of the pubes. The tumor was firmly adherent to all parts of the abdominal walls, and an attempt to pass the grooved director between the cyst and the peritoneum resulted in the rupture of the thin and lacerable walls of the tumor, and the escape of its fluid contents. The omental adhesions were extensive; but there was not adhesion to any other abdominal, nor to any pelvic viscus. The great mass of the tumor consisted of colloid matter, which, in order to remove through the wound in the abdomen, it was necessary to break down into hands-full. The mass being removed, and the pedicle, which was short and only moderately thick, tied by transfixion and double ligature, the abdominal cavity was cleansed, by sponges and salt water, of its colloid and cystic contents. The abdominal wound was closed by six equidistant silver wire sutures. The patient was then put to bed between woolen blankets; a number of hot bricks were applied, and alcoholic stimulants administered, to promote reaction. The wound was dressed with compress and bandage. Beef tea and brandy were continuously given per rectum, care being taken to avoid provoking movement of the bowels, by injecting slowly and in small quantities. The catheter was used regularly at intervals of six hours. After reaction was well established, the patient commenced to vomit, and vomited almost incessantly for three or four days. Morphine was given hypodermically, in quantities sufficient to allay pain and procure rest; and quinine was administered in the same way to the amount of about fifteen grains daily. Bismuth, in twenty-grain doses, and ice, allayed the irritability of the stomach sufficiently to enable the patient to take considerable quantities of milk. The convalescence of the patient has been much retarded by the old inflammatory disorders, and by an accumulation of putrescent fluid in the peritoneal cavity, which was limited by pelvic and abdominal adhesions. The presence of this fluid in the pelvic cavity gave rise to a general cellulitis, and the sufferings of the patient were further increased by the occurrence of cystitis.

Ten or twelve days after the operation, Dr. Bogue, in consultation with Drs. Fitch and Quine, drew off, through the cul-de-sac of Douglass, by means of the aspirator, nearly three pints of a grumous, fetid fluid, affording prompt and great relief to the pelvic distress. There has been no considerable reaccumulation of fluid, a spontaneous opening into the intestine affording ready drainage of the cavity. The treatment of the case has, in consequence of the complications, been widely different from the plan ordinarily followed, and combined measures directed to the cure of the old intestinal disorder, and the acute inflammation of the bladder. But notwithstanding the almost unprecedented desperateness of the case, and the numerous and alarming drawbacks to convalescence, the patient has been improving steadily but slowly, so that, at the present time, perfect recovery may be confidently predicted. I am requested by Dr. Quine to gratefully acknowledge his indebtedness to Mrs. Prosser, for her faithful and intelligent care of the patient, and to Drs. Bogue and Fitch for frequent and valuable counsels. But to the attentive lady nurse he especially ascribes the successful issue of the case.

Dr. Hutchinson gave a verbal report of two interesting cases of ovariotomy which he had had. He thought his cases were more successful than the others which had been reported, for, besides recovering without a bad symptom, each patient had become pregnant since.

Dr. S. J. Avery reported the following case: Sciatica of several years' standing, cured by replacing a prolapsed uterus. About the middle of August, 1871, he was called to see Miss T. He found the patient, a young woman, 21 years of age, of slight figure, a little below medium height, light complexion and nervous temperament, suffering with pain of right thigh and leg. The following history he learned from the patient: Some three years before, while employed as teacher in an academy in the State of New York, she was attacked with severe pain in the back and hips, extending down the thighs. During the following twelve weeks, she was confined to her room, and a large part of the time to her bed, being unable to move without assistance. She now began to improve in strength and was soon able to take short walks, but was not free from pain or lameness, from the first attack until the present time. During this period she had suffered several attacks similar to the first, lasting two or three weeks, confining her, as at the first, to her room and bed. She had also suffered, from the first, from dysmenorrhœa.

Upon learning her previous history, he informed the mother of the patient, a woman of more than ordinary intelligence, that he suspected uterine difficulty and probably displacement as the cause of her daughter's illness. She replied, that their old family physician, in whose judgment they had implicit confidence, as also, several other medical gentlemen in whose care she had been, had never suggested any difficulty of that nature. It was the desire of herself and daughter that electricity be tried, as that had been highly recommended. According to their wishes he applied the interrupted current—using one of Kidder's batteries—over the course of the sciatic nerve twice a week, for nearly three weeks, the patient taking bark and iron as a tonic. Under this treatment she seemed to improve, and experienced a hope that she was really recovering her health. About this time he was called in haste, the messenger saying, that Miss T. was in spasms. On his arrival he found her suffering from excessive dysmenorrhœa, which was soon relieved by the use of chloral and bromide of potash. One week after, upon making a digital examination, he found the vulva, beside being somewhat swollen, very tender and sensitive to the touch, the os resting upon the unruptured hymen, which was apparently the only obstacle to complete procidentia. He succeeded with some difficulty, on account of the swollen and tender condition of the parts, in placing the uterus in its normal position, and left the patient with directions to rest in the horizontal position as much as possible, promising to return in three days. On the third day following he found the patient much improved. She stated that she had been free from pain since his last visit. The day before, being Sunday, she had walked to and from church, a distance of nearly half a mile, without any inconvenience, and felt better than she had before since her first attack.

He again replaced the uterus, which had fallen about one-third the length of the vagina, and left, promising to call again in one week. He visited her twice after this, when he found the displacement so slight that further treatment was unnecessary. She took no medicine while receiving local treatment. The patient now returned to her home in the East. Fourteen months after, he received a letter from her, stating that she continued to improve, had gained sixteen pounds in weight, and that she considered her health restored.

The points of interest in this case, are, first, the importance of a correct diagnosis of the causes of backache and sciatica in the female; second, the *probability* of immediate relief in all cases of coincident retroversion by adjusting the womb; third, the possibility of the cure being permanent even without an instrument, care being taken to convert the *retro*version into an *ante*version in order to secure the antagonism of the normal intestinal pressure.

On motion, the discussion of this case was postponed until next meeting.

Dr. T. D. Fitch presented a new pessary which he had had constructed for the treatment of prolapsus, posterior and lateral version of the uterus. The instrument consists of the lever pessary similar to Noeggerath's, with a ring attached to the cervical end in such a manner that when the pessary is in position, the cervix rests evenly upon the ring. The advantages claimed by the Dr. for his instrument in the treatment of these displacements are, that it maintains the organ in position better, and affords increased surface for pressure, and is much more comfortable to the patient.

Owing to the lateness of the hour, the discussion of this was also postponed until next meeting. A cut and full description of the instrument will also appear in one of the medical journals.

A motion to make the hour of meeting 7½ o'clock, instead of 8, prevailed, and the Society adjourned.

